# Synchrony in triadic jumping performance under the constraints of virtual reality

**DOI:** 10.1038/s41598-022-16703-4

**Published:** 2022-07-20

**Authors:** Ayana Naito, Kentaro Go, Hiroyuki Shima, Akifumi Kijima

**Affiliations:** 1 Integrated Graduate School of Medicine, Engineering, and Agricultural Sciences in University of Yamanashi, Kofu, 400-8510 Japan; 2 Graduate School Department of Interdisciplinary Research in University of Yamanashi, Kofu, 400-8510 Japan; 3 Department of Environmental Sciences, University of Yamanashi, Kofu, 400-8510 Japan; 4 Faculty of Education, University of Yamanashi, Kofu, 400-8510 Japan

**Keywords:** Psychology, Human behaviour

## Abstract

The use of an immersive virtual reality system as a work space for sports and physical education can help maintain physical communication from separate places. In this study, we verified the possibility of constructing a movement synchrony system by reproducing the mathematical ordered pattern of “triadic jumping” in a virtual space. Three jumpers were asked to move together in a space that was cramped and insufficient for them to pass each other. Within this restricted space, the ordered pattern of the jumpers’ synchrony systematically transited to another state depending on the geometrical configuration of the work space. Although the temporal rigidity of the synchrony was partially lost, the ordered pattern of the “triadic jumping” synchrony that emerged in the virtual space was qualitatively equivalent to that emerging in real space. We believe the idea of expanding the work space for physical education to a virtual one could turn into reality if the sensory feedback of the collision successfully improves the spatial-temporal rigidity of the joint action ordered pattern.

## Introduction

Owing to virtual meetings and cloud storage, people across the globe are able to overcome communication limitations in terms of distance and availability. These communication technologies are especially important in the present state owing to the COVID-19 pandemic^1,2^. However, developing a virtual physical reality, for example, constructing an online dance hall that can virtually synchronize the moves of dancers who are not present in the same physical space, is challenging. To construct such an environment, the lag among individual actions must be suppressed to several tens of milliseconds. Additionally, synchronization patterns underpinned by a mathematical theory (i.e., self-organized patterns with temporal and spatial order, wherein the state transition is governed by the group theory as shown in Table S1) should be replicated in the space. In this study, we show that an ordered pattern of whole-body joint action can be robustly attained in a virtual space. Furthermore, we discuss the potency of virtual spaces as work space for sports or physical education, which can enable the emergence of a strict ordered pattern for body synchrony.

### Dynamical structure of the physical action synchrony

The limb synchronization patterns of legged animals can be analyzed as a nonlinear dynamical system, and their mechanism can be explained using the principle of nonlinear coupled oscillators^3–5^ or the theory of Hopf bifurcation (i.e., a type of bifurcation wherein periodic solutions occur owing to changes in system stability)^6–8^. For example, when two actors are asked to coordinate either one of their limbs or the whole body, their synchrony fluctuates when the frequency reaches a critical value, followed by a sudden phase transition^9,10^. The stability of the bifurcation patterns fluctuates depending on the inertial effects on the actor’s body or their direction of gaze^11,12^. These ordered patterns of dynamical systems can be applied to more complex joint movements in sports, such as dance^13,14^, 1-on-1 match up scenes in basketball^15,16^, and martial arts^17–20^. For example, Okumura et al. observed a spontaneous phase transition in the synchrony of the whole body movement of kendo players’ during the forward–backward step, which was equivalent to that of nonlinear coupled oscillators^17–19^. Yokoyama et al. analyzed the synchrony in the triadic joint action of soccer players based on the group theory and revealed a symmetry in the synchrony of Hopf bifurcation in excellent players^21,22^. Consequently, the ordered pattern of human synchrony is being unveiled based on the theory of symmetry breaking^23,24^.

Considering the aforementioned example in a dyadic system, under strict spatial–temporal constraints, either of the two actors instantaneously leads the other at a given moment. This leader-follower relationship alternates constantly. For example, in martial arts, if the distance between the fighters is sufficiently shortened, both fighters compulsively move forward or backward depending on the opponent’s choice^17,25^. The system is highly unstable and moves towards the end of the game when both fighters simultaneously move forward. Conversely, if both fighters move backward, the system remains stable but will halt, and the “dynamic” stability is lost. Therefore, both fighters share the 1D distance that enables them to hit each other, and the continuously fluctuating coordination dynamically stabilizes the tit-for-tat game state.

Similar synchrony occurs in more casual situations. For example, when two pedestrians cross a narrow path, both deviate from their walking trajectory in the same direction to pass through. Suppose you walk through a 2D space and have to cross two other people coming from different directions. If strict spatial–temporal constraints act on this action coordination, all three would deviate from their walking trajectory in the same direction without stopping each other. Therefore, the coordination of the direction between walkers *a* and *b* must be matched to those between both these walkers and the other walker *c*. Three constraints, namely the geometry of the work space (environmental constraint), ability of each walker (organismic constraint), and the spatio-temporal demands of the walkers (task constraint), can effectively ease this frustration^26–28^. Thus, the ordered pattern of movement coordination involving more than two elements can be reproduced by adopting an experimental system that enables the independent control all three constraints.

### Triadic jumping task

**Figure 1:**
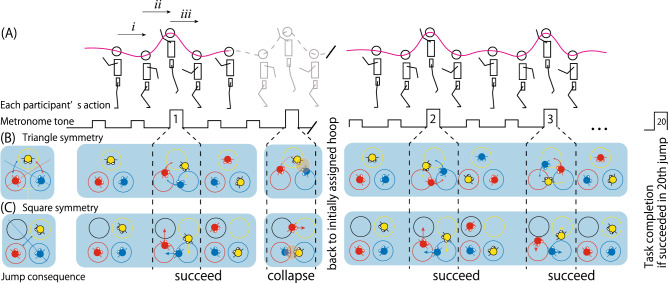
Triadic jumping task performed in the real and virtual spaces. (**A**) Jumping timing and jumper’s action sequence. All three jumpers are cued every 3 s by a high-pitched metronome tone following two low tones, as shown in the pulse diagram. The jumpers prepare for the high-pitched metronome tone by swinging their arms backward and bending their knees, as shown in phase *i*. Then, they swing their arms forward and upward and jump using ground reaction force earned during the movement in phase *i*, indicated as phase *ii*, followed by touch down phase *iii*. During these preparatory and jumping phases, the participants’ vertical head position fluctuates along with trajectory indicated in magenta. (**B**) Initial hoop-jumper configuration in the triangle geometry with $D_3$ symmetry is shown in the left-hand side. Regular triangle configuration is retained even if the configuration is rotated by 0 (360)$^{\circ }$, 120$^{\circ }$, or 240$^{\circ }$, and if the configuration is reversed about any of the three axes (colored in red, blue, and yellow; also see Supplementary Information). (**C**) Initial hoop-jumper configuration and the following sequence of failed and succeeded jumps in the square condition. Symmetry of the initial hoop-jumper configuration (left) is $D_1$ such that it can only be rotated by 0 (360)$^{\circ }$ and possesses only one reversing axis (colored in blue) to retain the initial hoop-jumper configuration. The jumping action sequence performed by three jumpers can be seen in the Supplementary Video S1.

Triadic jumping is an experimental system that enables control over the geometrical constraints of the joint action environment and eases the frustration caused due to triadic (3-actor) action coordination^29^. Actual performance of the triadic jump is shown in Supplemental Video S1. The triadic jumping work space is constructed by aligning three or four hoops (each $\phi$=0.65 m) on the laboratory floor, as shown in the left-hand side in Fig. 1B,C, respectively. In this study, three participants in a group were asked to stand in the assigned hoop and jump to the left or right hoops while receiving the auditory signal from the metronome. As shown in the pulse diagram in Fig. 1A, the jumping signal was presented following two warning tones once per second; therefore, three jumpers had to coordinate their jump direction and synchronize the jump timing at every 3 s. A trial ended when 20 successful jumps were recorded, to avoid the effect of participants’ fatigue while ensuring the statistical reliability of the mean and standard deviation of the timing of the jumping delay among the participants (i.e., $lag_\mathrm{mean}$ and $lag_\mathrm{sd}$, see “Dependent measures and statistical analysis” section under the “Methods” section). If the participants failed to synchronize their jump direction (by colliding with other jumper(s)), they were asked to return to their initially assigned hoop (for graphic explanation, see Fig. 1A).

**Figure 2:**
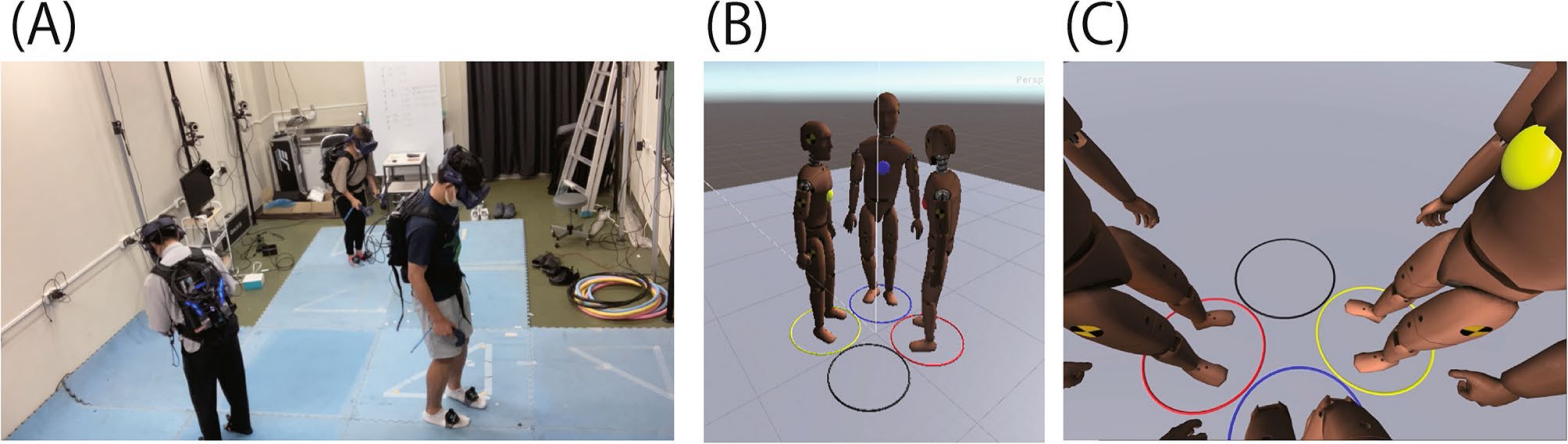
Work space of triadic jumping constructed in the virtual space. (**A**) Three jumpers in the laboratory immersing into the virtual triadic jumping space. Each was assigned a separate place so that they do not collide into each other. (**B**) Bird’s eye view of triadic jumpers used by an experimenter to check for collisions among the jumpers. Collision may occur only in the virtual space and cannot be confirmed in the real laboratory space. (**C**) Sight of a jumper (blue in the example) through the head-mounted display seeing the center of the square configuration of the 4-hoop alignment.

As shown in Fig. 1B,C, three jumpers had to jump to the same direction regardless of the hoop conditions (triangle or square). While this would be easy if the jumpers established mutual understanding about the jump direction, they were prohibited any exchange of verbal speech, glance, or other bodily expressions. Furthermore, they were prohibited from favoring a particular jumping direction before the trial began. Moreover, they were asked not to repeat the same jumping direction or style (e.g., left-right-left-right...). Therefore, the jumpers had to predict the jump direction by synchronizing the preparatory posture to jump left or right, as shown in the phase *i* in Fig. 1A. The three jumpers synchronized this preparatory action and gained sufficient ground reaction force to jump and move into the next hoop, which is approximately separated by 0.65 m. Despite such difficulty, the triads collided 3 to 4 times on an average until they completed 20 jumps in the previous study^29^, irrespective of the hoop alignment conditions.

**Figure 3:**
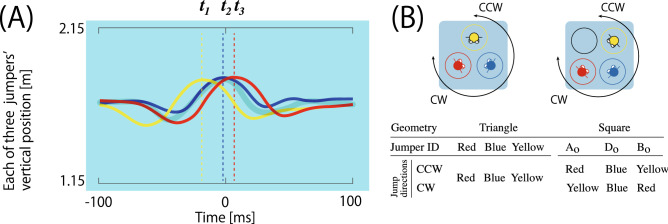
Vertical fluctuation of the jumper’s head and the definition of the (relative) positions of the three jumpers. (**A**) A typical example of the vertical head position of the three jumpers clipped from 100 ms before and after the peak of the averaged trajectory (shown by transparent gray trajectory). Each timing of the peak ($t_{1,2,3}$) was defined as the timing of the jumping action. Each jumper’s jumping action to generate this trajectory is illustrated in the image at the top of Fig. 1A. The pattern of vertical movement around each jump was identical for all three jumpers and within 20 jumps. (**B**) Definition of the jumpers’ positions. In the triangle condition, the probability to be a lead jumper is calculated for each of the three jumpers’ positions identified by the initially assigned hoop color: red, blue or yellow. In the square condition, the probability to be a leader is calculated for each jumper in each position relative to the open hoop, including *ahead*, *diagonal*, or *before* of the open hoop identified as $A_o$, $D_o$, and $B_o$, respectively. In the square condition, the jumper positioned in each of three positions differs according to the jumping direction, as shown in the table. Note that the red, blue, yellow, and black hoops were aligned in the same relative position for all seven triads, and the relative position of the three jumpers never changed considering they had to go back to the initially assigned hoop if they collided with each other (see, Fig. 1A–C, Supplementary Video S1).

All three jumpers’ degrees of freedom were determined by the hoop assigned to them. The spatial constraints for each jumper were symmetrical and asymmetrical in the triangle and square conditions, respectively. The constraint on each jumper was kept constant until the trial ended (i.e., the triad completed 20 successful jumps), considering the jumper returned to the hoop assigned when they failed to synchronize (see Fig. 1A). Under such spatial constraints, the jumper leading the other two jumpers was systematically emerged as the lead jumper in each jump. The lag of the other two jumpers was much shorter ($\approx$ 0.1 s) compared with the whole-body reaction time ($\approx$ 0.3–0.4 s) or the simple reaction time ($\approx$ 0.2 s). The probability of all three jumpers being the lead jumper was equal (i.e., 33.33% for all three jumpers) in the triangle geometry, whereas only the jumper standing in the hoop just before the open space in the jumping direction would be the lead jumper in the square geometry (i.e., $B_o$ in Fig. 3). The geometrical position of the lead jumper can be predicted by analyzing the geometrical hoop-jumper configuration based on the group theory. The details are explained in the Supplementary Information: “Analysis of the jumper-hoop configuration and the asymmetry in the jumpers’ role based on group theory” and Table S1.

**Figure 4:**
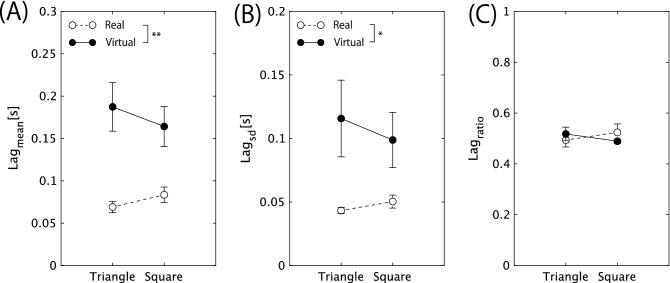
Mean and standard error of timing lag between the leader and followers. (**A**) $lag_\mathrm{mean}$, (**B**) $lag_\mathrm{sd}$, and (**C**) $lag_\mathrm{ratio}$. Broken line with open circle denotes real space, and the solid line with filled circle denotes virtual space. ** and * indicate a significant difference with $p< 0.01$ and $p< 0.05$, respectively.

Therefore, the geometrical structure of the jumper-hoop configuration constrains and eases the frustration of the jumping direction emerging in the synchrony of the three jumpers. Consequently, both *quantitatively* and *qualitatively* strict spatio-temporal patterns emerge under such a systematic constraint, which are physically accurate and reproducible at a statistically significant level. Thu, the triadic jumping is the only system that enables the reproduction of such an ordered pattern of the triadic action synchrony.

**Figure 5:**
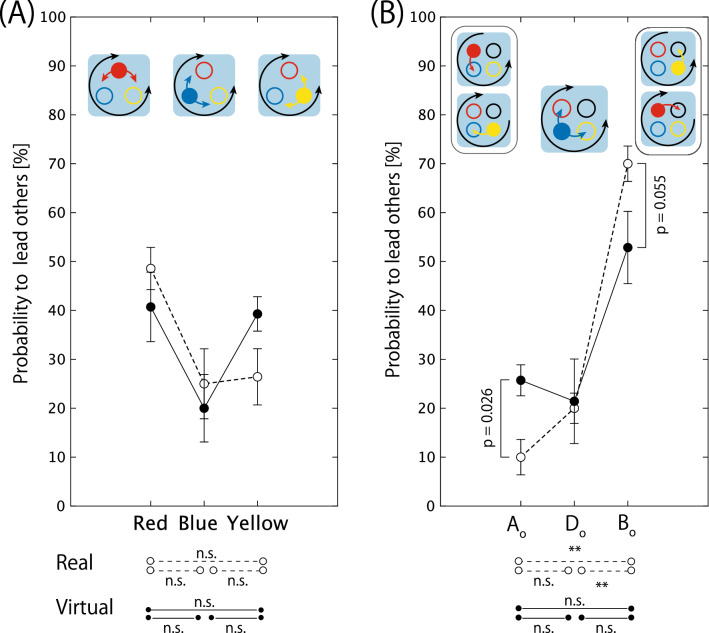
Probability of being a lead jumper (in percentage) relative to 20 successful jumps. Each probability is calculated for each jumper assigned to each of the three positions differently defined in each geometrical condition (see Fig. 3B for detail). Mean and standard error of seven triads. (**A**) Triangle geometry comprising three positions defined by red, blue, and yellow hoops and (**B**) square geometry comprising four positions defined by the relation to the open space and jumping direction; open space, one hoop ahead from open ($A_o$: Red for counterclockwise and yellow for clockwise), diagonal to open ($D_o$: Blue for both directions), and one hoop before open ($B_o$: Red for clockwise or yellow for counterclockwise) each with respect to the direction of the jump. Broken line with open circle denotes real space, solid line with filled circle denotes virtual space. **indicates a significant difference with $p< 0.01$ in the post-hoc test using the Bonferroni method.

### Physical synchrony of triadic interpersonal action in virtual reality

This study replicates the physics of real-world action synchrony based on the principles of dynamical systems and clarifies that an ordered pattern of action synchrony emerges not only in a physically real space but also in an immersive virtual reality space.

Many previous studies on “synchrony” in virtual space consider synchrony to be a phenomenon wherein prosocial emotions emerge in the cooperation with avatars^30,31^. These studies emphasized the fact that such prosocial emotions are not by-products of the intention or togetherness to maintain synchrony but emerge in the “unintentional motor synchrony” with the avatar^32,33^. However, to date, only a few researcher^34^ has demonstrated the reproduction of the “dyadic (2-actors)” action synchrony that unintentionally transits to different states under physical constraints without being instructed by the experimenter to synchronize with a partner. Against this backdrop, we replicated an experimental system on a virtual space: triadic jumping that induces and emerges synchrony among three actors by applying geometric constraints to the virtual work space without cognitive intervention in the participant’s intention. Furthermore, we calculated the temporal and spatial measure of the synchrony to compare the coherence of the ordered pattern emerged in the virtual space with that in the real space.

A virtual reality system is expected to be an experimental tool that enables the reproduction of complex daily movements while controlling the visual field of the work space^35–38^. It has been reported in the previous research that the physical quantity of the movement in the virtual space is not exactly same as that in the real space (e.g., a decrease in the movement’s amplitude^38,39^ results in an increase in the distance between two pedestrians crossing a road^40–42^). However, the reproducibility of the qualitatively ordered pattern of triadic action synchrony has not been clarified, and the possibility of expanding the work space for the synchrony of human action to the virtual space remains unknown. Wherein, we investigate the possibility of realizing physically ordered synchrony of perceptual-motor behavior in the virtual space. In our daily life, we maintain our physiological and mental health by spontaneously cooperating with peers who are physically close to us. Therefore, if the ordered pattern can be reproduced with the same accuracy as that in real space, it may be possible to reproduce the positive health effects caused by the action synchrony in the virtual environment even for a person who is physically separated. Thus, the system could enable the creation of a virtual playfield and bring about physiological and psychological well-being considering it draws more attention in the current state of mental and physical health risks due to COVID-19.

## Methods

### Participants

Fourteen participants were recruited among students of the Department of Physical Education at the University of Yamanashi. Seven triads were composed, with the seven out of 14 participants involved in the two triads. Four participants were female (Age = 18.00 ± 0.00 years, Height = 160.50 ± 5.20 cm, Weight = 53.75 ± 6.50 kg), and ten were male (Age = 20.40 ± 1.51 years, Height = 171.80 ± 6.12 cm, Weight = 68.40 ± 11.75 kg). Two out of the four female participants were involved in two triads, while the other two were involved in one triad. Any effects arising out of gender distribution were not observed in the results. All participants possessed good physiological and psychological health and had no experience of triadic jumping in both real and virtual spaces. This study was approved by the Research Ethics Committee of the Faculty of Education, University of Yamanashi and was conducted in accordance with the Declaration of Helsinki. Moreover, all participants provided informed consent before enrollment. All experiments were performed in accordance with approved guidelines and regulations.

### Real work space setting

#### Procedure

Urethane foam mattresses were placed on the laboratory floor, and three or four plastic hoops (each $\phi$ = 0.65 m) were aligned in regular triangle or square shapes, respectively. The position of each hoop in each of the triangular and square geometries was aligned as shown in the left-hand side in Fig. 1B,C, respectively. The red and yellow hoops were aligned to the left- and right-hand sides of the blue hoop, respectively, whereas the black hoop was aligned diagonal to the blue hoop. Three participants in each triad were asked to remove their shoes and stand in each hoop with a randomly pre-assigned color. A rhythmic tone was presented and detailed instructions about the task were given to the participants. Subsequently, we asked them to jump once during the high tone that cyclically occurred every 3 s following two warning signals, each presented by a low tone, as shown in Fig. 1A. We confirmed in advance that an interval of 3 s is sufficient for the participants to maintain their posture while preparing for the next jump. If the participants missed a synchronization or collided with each other, they were asked to return to the pre-assigned hoop. A trial continued until 20 successful jumps were achieved. Thereafter, they were asked to perform triadic jumping in either geometrical condition that is different to the conditions they already completed. In case they missed a jump and returned to the assigned hoop, the experimenter verbally present the signal to restart the jumping trial (e.g., “So, lets prepare for the next signal, three, two, one... go!”). The typical situation in both the triangle and square geometries is shown in Supplementary Video S1. The three jumpers in the video have approved the video to be published online.

#### Hardware setting for measuring jumping movement in real space

Before the beginning of the trial, all three participants wore a motion capture cap on their head. Three reflective markers were attached to each participant’s head. The positions of the nine markers were recorded using 15 infrared motion capture cameras (Miqus M1, Qualisys Inc., 100 Hz). Furthermore, the 3D positions of the three markers on the heads of each of the three participants were calculated, whereas the median point of the three markers was calculated as the position of the top of each participant’s head.

### Virtual work space setting

#### Procedure

Three or four days after the completion of the trial in the real space, each triad jumped in both of triangle and square geometry constructed in the virtual space. The urethane mattress was sufficiently wide to avoid any collision among the three jumpers as shown in Fig. 2A. The experimental system was constructed in the same room as that of the real space, and the mattress was the same as that used in the trials in the real environment. The participants were asked to wear a head-mounted display (HTC VIVE Pro Eye, HTC Inc.) and hold a controller in their hands. Their dorsum and waist were attached to a VIVE tracker. The participants could move freely in the tracking space, which was separately set for each jumper, and each of them was immersed in the virtual triadic jumping space, as shown in Fig. 2A. The virtual triadic jumping space was shared with the other two jumpers using a backpack PC (VR GO 2.0, ZOTAC Inc., intel^®^Core i7-8700T processor NVIDIA GeForce^®^GTX 1070 graphic) wired to the head-mounted display (HMD). The work space of the triadic jumping was projected to each jumper via a head-mounted display, which was qualitatively the same as the real work space. Each jumper was assigned to one of the three hoops marked in red, blue, and yellow. The metronome tone, which was adopted to instruct the timing of the jump, was the same as that used in the real space. After all trials under the triangle and square conditions were completed, all participants separately answered a questionnaire concerning their age and physiological profiles (sex, height, weight, and sports experience), prior experience of virtual activity (such as playing games or watching movies), feeling with disturbance owing to the equipment in contact with their body, and the ease of the task in the real and virtual spaces.

#### Hardware and software configuration for constructing a virtual work space

We used the Lighthouse System (HTC Inc.) to track the head-mounted display, tracker, and controller positions. We set four base stations that segregated the tracking space into three divisions, each assigned to a participant. Considering the triadic jumping game requires three players, each player wore a backpack PC with an HMD, two controllers (for each hand), and three trackers (for both the foots and the waist). The backpack PC collected the position of each device to track the player’s head, hand, foot, and waist positions. Then, the backpack PC transferred the position data to a desktop PC (Intel Core i7 7700 K 4.20 GHz), which collected the data on the position of the body parts of the three players. Finally, the desktop PC constructed a virtual space for a triadic jumping game using Unity (Version 2018.2.11f1) with three virtual avatars that corresponded to the three players. We placed three or four hoops in the virtual space in the exact same setting as that in the real space, as shown in Fig. 2B (i.e., the hoop $\phi$ = 0.65 m and aligned as their edges touched each other). Figure 2B shows the three avatars, each of which is placed in a hoop in the virtual space. We controlled the avatars in the virtual space using the inverse kinematics algorithm package, which is designed for Unity (Root Motion Inc.), based on the positions of the body parts of the players. Additionally, we used a network engine service (Photon Unity Networking 2, PUN2) to deploy a multiplayer game with three avatars. Figure 2C shows the player’s view of the replicated task space.

### Dependent measures and statistical analysis

The vertical position of each jumper’s head was obtained as data in the real and virtual work spaces. To detect the timing of a jump, the time series of the three jumpers’ positions were averaged, and the peak timing was assigned as the timing of each jump. The transparent plot in Fig. 3A shows an example of the averaged time series in a typical jumping action and the fluctuation of the position of each jumper’s head, measured during the same jumping action. The timing of all three peaks was distributed at the peak of the averaged time series. As shown in Fig. 3A, the timing of the peak at which the lead jumper performed the $n\text{th}$ successful jump is defined as $t_1(n)$, and those for the $2\text{nd}$ and $3\text{rd}$ jumpers are defined as $t_2(n)$ and $t_3(n)$, respectively. The representative value of the two followers’ delays in the $n\text{th}$ jump was calculated as:

1 $$\begin{aligned} L_n = \frac{\sum ^{3}_{i=2}(t_i(n)-t_1(n))}{2}. \end{aligned}$$

This representative value for each of the 20 successful jumps, $L_1$, $L_2$... $L_{20}$, was calculated, and their mean and standard deviation are defined as $lag_\mathrm{mean}$ and $lag_\mathrm{sd}$, respectively. Moreover, the ratio of the lag of the $2{\text {nd}}$ jumper to that of the $3{\text {rd}}$ jumper was calculated for each of the 20 successful jumps, whereas the mean of these values was defined as $lag_\mathrm{ratio}$. Two-way ANOVA, with repeated measurements comprising the task space (2: real, virtual) and hoop condition (2: triangle, square), was applied to all three variables. The level of statistical significance was set to $p< 0.05$.

As shown in Fig. 1A–C, the relative relationship of the three jumpers’ position does not change until they complete 20 successful jumps, considering each jumper had to return to the initially assigned hoop when they missed to achieve synchrony (see, Supplementary Video S1). Furthermore, there was no difference in the degrees of freedom among the three jumpers assigned to the red, blue, or yellow hoops in the jumper-hoop configuration of the triangle geometry shown in Fig. 1B and left-hand diagram of Fig. 3B. Therefore, in the triangle condition, we simply calculated the probability of a participant being the lead jumper initially assigned to the red, blue, or yellow hoop. Contrastively, in the square condition, a jumper initially assigned to the red and yellow hoops has an open space (depicted by the black hoop in the right-hand diagram of Fig. 3B) in the left and right, respectively, whereas the jumper assigned to the blue hoop does not have any adjacent open space. Because of this asymmetry, the probability of each jumper being the lead jumper was not simply defined with respect to the absolute position of the hoop defined by those colors; instead, it was calculated based on the position relative to the open space, which was dependent on the direction of each jump, as shown in right-hand diagram and the table of Fig. 3B.

- $A_o$ A jumper assigned to one hoop *ahead* of the open hoop (i.e., yellow and red colored jumper for clockwise and counterclockwise direction, respectively).
- $D_o$ A jumper assigned to the hoop *diagonal* to the open hoop (blue colored jumper for both directions).
- $B_o$ A jumper assigned to one hoop *before* the open hoop (i.e., red and yellow colored jumper for clockwise and counterclockwise direction, respectively).

Thus, the probability of being a lead jumper was calculated for each jumper in each of the three positions differently defined in each geometrical condition. The statistical significance of the effect of task space (2: real, virtual) and hoop position (3: red, blue, and yellow for the triangle condition; 3: $A_o$, $D_o$, and $B_o$ for the square condition) and their interaction on the probability of each jumper being the lead jumper was tested using a two-way ANOVA with repeated measurements. The multiple comparison for the (simple) main effect of the lead jumper’s position was tested using the Bonferroni method. The level of statistical significance was set to $p< 0.05$

## Results

### Jump timing lag and jump direction between the leader and followers

The lag between the lead jumper and the followers was calculated based on the vertical head position of the jumpers and the mean of the representative value of the leader-follower lag (for details of the calculation procedure, see Fig. 3A and Eq. (1)), which was calculated for each of the 20 successful jumps and averaged as $lag_\mathrm{mean}$. While the main effect of the task space was statistically significant (*F*(1, 6) = 19.675, *p* = 0.004, $\eta ^2$ = 0.766), the main effect of the hoop alignment (*F*(1, 6) = 0.427, *p* = 0.537, $\eta ^2$ = 0.066) and the interactive effect of both factors (*F*(1, 6) = 3.09, *p* = 0.129, $\eta ^2$ = 0.34) were not significant. Furthermore, the mean of the $lag_\mathrm{mean}$ averaged over the seven triads was significantly shorter in the real space (0.076 s) than in the virtual space (0.176 s). The value observed in the real space was extremely short compared with the response time of the whole-body coordination. Although the $lag_\mathrm{mean}$ measured in the virtual space was significantly longer, the value was significantly shorter than the whole-body reaction time ($\approx$ 0.3 s; the interval between the trigger signal to jump to the moment of the foot’s take off) measured in the same age group^43^. Moreover, it was even shorter than the simple reaction time ($\approx \,0.2$ s). Additionally, as shown in Fig. 3A, all three jumpers’ actions, including the preparation to takeoff, constantly fluctuated in the isomorphic (Mexican hat) shape. Thus, the two followers did not *react* to the leader’s jump direction, but instead, all three jumpers synchronized their preparatory action and *mutually interacted* to extrude jumpers who led others towards the jumping direction.

Furthermore, we calculated the standard deviation of the leader-follower lag of 20 successful jumps ($lag_\mathrm{sd}$) for each trial. The two-way within-group ANOVA revealed a significant task space effect (*F*(1, 6) = 6.95, *p* = 0.038, $\eta ^2$ = 0.537) on the $lag_\mathrm{sd}$, whereas the main effect of the hoop alignment (*F*(1, 6) = 0.697, *p* = 0.435, $\eta ^2$ = 0.104) and the interaction of the task space and the hoop alignment (*F*(1, 6) = 1.846, *p* = 0.223, $\eta ^2$ = 0.235) were not significant. As shown in Fig. 4B, the mean of the $lag_\mathrm{sd}$ averaged over seven groups measured in the real space (0.047 s) was smaller than that measured in the virtual space. Additionally, as shown in Fig. 4B, the between-triad deviation was smaller for the real-space condition. The results show that temporal synchrony of the triadic jumpers are remarkably stable in the real space, whereas in the virtual space, the occurrence of temporal synchrony fluctuate stochastically. Moreover, we calculated the ratio of the $2{\text {nd}}$ jumper’s lag to the third jumper’s lag ($lag_\mathrm{ratio}$). The results shown in Fig. 4C suggested that the main effects of the task space and the hoop alignment, and their interaction, were not significant (task space: *F*(1, 6) = 0.055, *p* = 0.823, $\eta ^2$ = 0.009; hoop alignment: F(1,6) = 0, *p* = 0.983, $\eta ^2$ = 0.000; interaction: *F*(1, 6) = 1.603, *p* = 0.252, $\eta ^2$ = 0.211). The value distributed around 0.5 indicates that the lag between leader and $2{\text {nd}}$ jumper and that between the $2\text {nd}$ and $3\text {rd}$ jumpers were equivalent. This trend was also observed for the trials in the virtual space.

In addition to the analysis of the temporal measure, we analyzed the jumping direction using three-way ANOVA with repeated measurements, which includes the task space (2: real or virtual), hoop alignment condition (2: triangle or square), and the jumping direction (2: clockwise or counterclockwise). As shown in Table S2 in Supplementary Information, in five out of seven triads, the number of jumps in the counterclockwise direction was more than that in the clock wise direction; however, the main effect of the jumping direction was slightly short compared with the statistically significant level (F(1,6) = 5.899, $p < 0.051$, $\eta ^2$ = 0.496). This trend was also observed in the previous research^29^. One may infer that this preference of jumping direction would be caused by the (right) hand dominance of the jumpers, however, no left-handed jumper was included in the 2 triads those preferred clockwise jumping. Additionally, statistical significance of the main effects of the task space condition and hoop alignment condition on the number of coordination miss (collision) was tested using two-way ANOVA with repeated measure. The results showed that although there was no significant main effect nor interaction. However, the overall number of collisions in this study was more than that observed in our previous research^29^ (Mean ± SD for each of four condition: 5.571 ± 3.156 for triangle condition in real space, 5.714 ± 2.373 for square condition in real space, 6.571 ± 4.136 for triangle condition in virtual space and 4.143 ± 1.457 for square condition in virtual space). This increase in the number of collisions can be attributed to the instruction in the current experiment, which prohibits jumping in the same direction repeatedly compared with the previous research.

### Geometrical position of the lead jumper

The jumpers were students in the same department of physical education and possessed the same body movement skills to perform triadic jumping. In the triangle condition, the degrees of freedom for the three jumpers were symmetrical (or equivalent), considering none of them had open space to move freely. We calculated the probabilities of each of the three jumpers assigned to the red, blue, and yellow hoops, to lead others. The two-way ANOVA with repeated measurements indicated no significant main effects of the task space (*F*(1, 6) = 0.277, *p* = 0.617, $\eta ^2$ = 0.044) and the hoop position (*F*(2, 12) = 2.760, *p* = 0.103, $\eta ^2$ = 0.315) and no significant effect of interaction (*F*(2, 12) = 3.546, *p* = 0.062, $\eta ^2$ = 0.371). This equivalence in the probability of being a leader shown in Fig. 5A can be predicted from the symmetrical configuration of the hoop-actor geometrical alignment, and this result suggests that the geometry constructed in the virtual space also constrained the ordered pattern of action synchrony among the three jumpers.

In contrast to the triangle hoop condition, there exists a “unique” open space in the square hoop condition and the degree of freedom of the three jumpers is asymmetrical (or non-equivalent) depending on the relative position to the open space that constrains each jumper’s degree of freedom until the completion of the trial. Considering both have open spaces on either side, the degrees of freedom for the jumpers initially assigned to the red or yellow hoop are symmetrical. By contrast, the degree of freedom for the jumper who was initially assigned to the blue hoop is not symmetrical to the other two, considering both sides of the blue jumper were occupied by the red and yellow jumpers. Based on group theory (see, Table S1), we predicted that such asymmetry in the geometrical configuration constrains the leader-follower asymmetry. To confirm this hypothesis, we compared the probability between the jumper’s position relative to the “unique” open space that introduces geometrical asymmetry into the system as explained in Fig. 3B.

As shown in Fig. 5B, the two-way ANOVA with repeated measurements revealed that the main effects of the task space were not significant (*F*(1, 6) = 0.151, *p* = 0.710, $\eta ^2$ = 0.016), whereas the main effect of the hoop position (*F*(2, 12) = 21.696, *p* = 0.000, $\eta ^2$ = 0.783) was significant. The result of the post-hoc test using the Bonferroni method indicated that the probability for the $B_o$ jumper (i.e., the jumper jumped from $B_o$ position) was significantly higher than that for the other two jumpers ($B_o$ vs. $A_o$ = 0.000, $B_o$ vs. $D_o$ = 0.000, $A_o$vs.Do = 0.128). The effect of interaction between the factors was also significant (*F*(2, 12) = 4.146, *p* = 0.042, $\eta ^2$ = 0.409). The analysis of the interaction revealed that the simple main effect of the task space was significant at $A_o$, whereas the probability in the virtual space was significantly higher than that in the real space (*F*(1, 6) = 13.444, *p* = 0.026, $\eta ^2$ = 0.771). The probability of $B_o$ was lower in the virtual space (*F*(1, 6) = 5.629, *p* = 0.055, $\eta ^2$ = 0.484); however, this tendency was slightly short to statistically significant level. The simple main effect of the hoop position in both task spaces was significant (real: *F*(2, 12) = 57.867, *p* = 0.000, $\eta ^2$ = 0.905; virtual: *F*(2, 12) = 4.171, *p* = 0.042, $\eta ^2$ = 0.410). However, the result of the post-hoc test using Bonferroni revealed that the difference between the hoop position was significant only in the real space, which indicated that the probability of the $B_o$ jumper was higher than that of the other two jumpers (*p* = 0.000 for $B_o$ vs. $A_o$ and $B_o$ vs. $D_o$, *p* = 0.128 for $A_o$ vs. $D_o$). Furthermore, there was no significant difference between the hoop positions in the virtual space. The result showed that the asymmetrical role of the jumpers’ position robustly reflects itself in the synchrony of the triadic jump, as predicted by the geometry of the hoop-jumper configuration. Conversely, in the virtual space, although the result of the post-hoc test was not statistically significant, the simple main effect representing an overall deviation among the three hoop positions was statistically significant. Furthermore, the statistically significant effect of the hoop position on the data combined with those measured in the real and virtual spaces were equivalent to the simple main effect in the real space only. Therefore, we could reproduce the asymmetrical ordered pattern of the triadic jumping in the square geometry in the virtual task space with a finite level of probability.

Additionally, according to the answers received for the questionnaire distributed after the experiment, seven participants had a better experience in the real space, while the other five had a better experience in the virtual space. The remaining two participants reported no significant difference in the ease between the two spaces. Two participants experienced severe disturbance in the virtual space, both of them reported the difficulties in understanding other jumpers’ intention.

## Discussion

In the real space, three jumpers synchronized with an extremely short phase lag ($lag_\mathrm{mean}< 0.1$ s), as shown in Fig. 4A. Moreover, the extremely small $lag_\mathrm{sd}$ in Fig. 4B indicates that the extremely short phase lag was stably reproduced for every 20 successful triadic jumps. In the square hoop condition, the constraint of the geometrical symmetry inherent in the triadic jumping system compelled the jumper in one hoop before the open space to lead the other two jumpers, resulting in the spatial-temporal ordered pattern shown in Fig. 5B. The ordered pattern of the triadic jumping emerges under the constraint of the geometrical parameters of the environment, task demand, and the ability of the participant to perform the task; it was not a product of intentional cooperation through language but emerged spontaneously and unintentionally under the geometric constraints of the system. Generally, in a system of action synchrony, the ordered pattern with the highest symmetry emerges among the patterns inherent in the system. This rule of synchrony has been repeatedly confirmed in many laboratory experiments^9,11^ and in the field of sports^17,21,25^. An emphasis should be placed on the fact that the ordered pattern of triadic jumping is endowed with mathematical rigor. In the triadic jumping system, a change in the hoop-jumper geometry prompted a bifurcation to states with different symmetry without the intention of the participants ($D_3$ for a triangular arrangement, and $D_1$ for that of a square arrangement; see Fig. 1A, Table S1 in Supplementary Information). This physically robust bifurcation would be unchanged even if the players were not always occupating the same hoop color (that is, their relative positions may change if they missed to synchronize), owing to the physical or mathematical law of the action synchrony or affordance^44^ perceived by each jumper who has or does not have open space next to her or him.

The most important result of this study is that the spontaneously emerging ordered pattern of action synchrony can be reproduced in the virtual space as well. Although the lag and its standard deviation were significantly larger in the virtual space compared with the data obtained in the real space, the ordered pattern that spontaneously emerged in our virtual system was far more stable and mathematically rigor compared with the synchrony in the previous virtual space research. For example, in the previous research^30^, the joint action between the avatar and the actors was considered to be synchronized if the lag was less than 0.60 s, which is far more longer than that in the triadic jumping action synchrony in our virtual space (i.e., $\le$ 0.2 s, as shown in Fig. 4A). Nonetheless, the temporal pattern inherent in the real physical world was actually perturbed by certain intrinsic factors of the virtual environment, which was also reflected on the qualitative order of the leader-follower interaction that emerged in the asymmetrical jumper-hoop configuration in the square geometry, as shown in Fig. 5B. These perturbations provide us with a profound insight on the “synchrony” of the human body action emerging in the physical world. The unique characteristics of the synchrony of the triadic jumpers is its temporal precision and rules following the mathematical law of the group theory. In this case, what sense would be needed for the actors to construct such a synchrony that involves an extremely short lag and a physically rigorous ordered pattern?

Vision impairment due to the HMD would be one of the possible distractor, considering the visual information primary mediates the real word interpersonal action synchrony^9,11,45^. However, the problem on the vision due to HMD would not completely explain the distraction on the synchrony. In fact, according to the answers given in the questionnaire, six out of 14 jumpers felt it was more difficult to “see” the other jumpers’ preparatory actions in the virtual space than in the real space. However, the refresh rate of HTC VIVE Pro Eye is 60 Hz ($\approx$ 16.7 ms/frame), and the interval between holding the key of the hand-held controller down to reflecting the result on the display was up to only 15 ms^46^. Moreover, even when the delay due to rendering a scene on the display accumulated, the total temporal delay from the jumpers’ action to the moment at which the result of the action visually reflected on the HMD was several tens of milliseconds. Therefore, it would not be reasonable to deduce that such a short delay would completely explain the subjective difficulty in the perceptual-motor act of the six jumpers. In fact, the delay in feedback sufficient to skew the sense of agency or body ownership can be postulated to be approximately 150–200 ms^47–50^. Additionally, it is reasonable to infer that the equipment’s heavy weight would disturb the participant’s jumping action. However, no participant reported difficulty due to the weight of the HMD, backpack PC, or other parts of the virtual system, including the VIVE tracker and hand-held controller. Therefore, although equipment weight will severely affects participants who have less physical strength (e.g., children), it did not have much influence on the actions of young participants in the current experiment.

Instead of such vision impairment, we propose the “physical” constraint, that is, the absence of the risk of a physical collision, as the main destructor to disturb emergence of ordered action synchrony. In fact, the lack of significance in the result of the post-hoc analysis of the virtual space data can be explained by two significant simple main effects: the increase in the probability of jumper $A_o$ (a jumper jumped from the hoop ahead of the open space in the jumping direction) leading the others, and the decrease in that of jumper $B_o$ (a jumper jumped from the hoop before the open space) leading the others compared with the probability in the real space. Each of the triadic jumpers swung their arms back and squatted downward in the preparation phase of the jump to gain a sufficient ground reaction force to jump (see, phase *i* in Fig. 1, Supplementary Video S1). This synchrony is necessary to coordinate their timing to the jump in both the real and virtual spaces. Furthermore, each jumper performs this action mutually adjusting their jump direction, and concurrently, mutually avoiding to touch other jumpers. Owing to this mutual posture control, the three jumpers were entrained to jump in the same direction, especially in the real physical world that involves physical risk of body contact. On the other hand in the virtual space, the intention to not touch other jumpers’ bodies does not strictly constrain their preparatory action synchrony considering that the jumpers are aware that they will not experience a touch sensation even while colliding with the body of another avatar. The absence of such a predictive sensation disturbs the spatial and temporal order of the physical synchrony. This perturbed coupling led to large $lag_\mathrm{mean}$ and $lag_\mathrm{sd}$, and also increased the probability of jumper $A_o$ leading to jump into the space already occupied by another jumper (i.e. $D_o$ or blue jumper in Figs. 3B, 5B). This hypothesis about the effect of the “physical” risk of collision on the stability of the ordered pattern can be tested by introducing the force feedback function to the current system.

Nonetheless, the synchrony in our virtual space is rather more rigid than that in the previous studies on virtual reality, and the absolute lag value was rather shorter than the simple reaction time. Furthermore, the jumper initially assigned to one hoop before the open space (i.e., $B_o$ jumper, see right diagram of Figs. 3B, 5B) led the other jumpers. Therefore, there is the possibility of systematically controlling the patterns of the physical synchrony in the virtual reality system by altering the geometrical layout of the work space. We could hypothesize about the important perceptual-motor act required to construct physically stable and mathematically rigorous synchrony, that is, the mutual regulation of “not-to-touch” distance. This perceptual-motor act could not be completely reproduced in our virtual reality system, and the missing part could be the *embodied* prediction of the touch with other jumpers. If a touch sensation feedback system fills this gap, we could create a virtual playground for physical education or sports that physically bonds people in separate places.

The COVID-19 pandemic has reiterated the need to develop such technologies to exploit their full potential. According to the research on health under high-risk situations for virus infections, the cognitive bond using online meeting systems decreases the feeling of loneliness and enhances the mental health of people lacking in-person communication^1^. Additionally, physical interaction has positive psychological effects, such as reduced depression^2^, which is equally important as prosocial emotions, as investigated in the research on the synchrony of humans and virtual agents. Based on these scientific findings and the knowledge learned from our experience, this effect of “physical” interaction should attract more attention, especially now in the high-risk situation of virus infections.

## Supplementary Information

Supplementary Information.

Supplementary Video S1.

## Data Availability

The datasets used and/or analyzed during the current study are available from the corresponding author on reasonable request.
