# Supplementary material for: Synchrony in triadic jumping performance under the constraints of virtual reality
Source: Sci Rep. 2022 Jul 20;12:12417. doi: 10.1038/s41598-022-16703-4 (PMC9297677; doi:10.1038/s41598-022-16703-4)
Supplement: Supplementary file 1 — Supplementary Information. [file 41598_2022_16703_MOESM1_ESM.pdf]

Supplementary information

Analysis of the jumper-hoop configuration and the asymmetry in the jumpers' role based on group theory

The shape of the regular triangle hoop alignment shown in the left-hand side in Figure **??**B remains unchanged even when rotated around either of the three axes (red, blue, and yellow). Moreover, it remains unchanged even when rotated over three angles: 0° (360°), 120°, or 240°. Therefore, the symmetry of the shape is defined as a dihedral symmetry D_3, based on the group theory. Conversely, the jumping ability of the jumpers is equivalent, and this functional symmetry can be defined as S_3. The isotropic subgroup of the hoop geometrical symmetry and the functional symmetry of the jumpers is shown in Table S1, where the maximum ordered pattern subgroup is D_3 (or S_3), which indicates that all three elements commute with one another, that is, the elements are symmetrical and correspond to the phenomenon wherein all three jumpers become leaders with the same probability.

Table S1. Isotropic subgroups of the jumper-hoop configurations.

Geometry	Hoop symmetry	Jumper symmetry	Jumper-hoop symmetry (isotropy subgroup)
Triangle	D_3	S_3	D_3 (or S_3), Z_3, D_1 (or Z_2), I
Square	D_4	S_3	D_1 (or Z_2), I

Conversely, the square shape of the hoop shown in the left-hand side in Figure **??**C is invariant to the reflection around the four axes (three dashed axes and the blue axis) and is invariant to the rotation over four angles: 0° (or 360°), 45°, 90°, and 135°. Therefore, the symmetry of the square hoop alignment is defined as D_4. Considering that the functions of the three jumpers are the same as those in the triangle condition, the symmetry can be defined as S_3. Therefore, the highest-ordered pattern of the isotropic subgroup of the hoop-actor symmetry can be defined as D_1 (or Z_2), which corresponds to the result of a jumper in the hoop before the open space with respect to the jumping direction, who can be a lead jumper. Only two out of three jumpers can be permutated. Subsequently, the symmetry of the jumpers' role can be predicted using the symmetry based on group theory, and the symmetry of their role is congruent with the highest-ordered pattern of the isotropic subgroup hoop geometrical symmetry and the functional symmetry of the jumper, that is, the hoop-jumper symmetry. For a more detailed explanation, see[1,2].

Jumping direction in the real and virtual space

Table S2 shows the sequence of the jumping directions of 20 successful jumps performed by each of the 7 triads (ID: t_{1-7}). The statistical significance of the task space condition (2: real or virtual), hoop alignment condition (2: triangle or square), and the directions of the jump (2: clockwise or counterclockwise) on the number of the jumps was tested using three-way ANOVA with repeated measure. Although there was a significant effect or the interaction between the three factors, the main effect of the jumping direction was slightly shorter than the significant level ($F(1,6)=5.899$, $p < 0.051$, $\eta^2=0.496$), which indicated that the triads jumped more in the counterclockwise direction than the clockwise direction. This trend was also observed in a previous study on triadic jumping[2].

Supplementary Video

Five triadic jumping actions in each of the triangle and square conditions performed by a typical triad.

References

1. Richardson, M. J. *et al.* Self-organized complementary joint action: Behavioral dynamics of an interpersonal collision-avoidance task. *J. Exp. Psychol. Hum. Percept. Perform.* **41**, 665 (2015).

2. Kijima, A., Shima, H., Okumura, M., Yamamoto, Y. & Richardson, M. J. Effects of agent-environment symmetry on the coordination dynamics of triadic jumping. *Front. Psychol.* **8**, 3, DOI: 10.3389/fpsyg.2017.00003 (2017).

Table S2. Directions of 20 successful jumps in the triangle and the square condition performed in the real and the virtual space. W: clockwise jumping. C: counterclockwise jumping. ID: triad ID. N_W: Number of jumps in the clockwise direction. N_C: Number of jumps in the counterclockwise direction.

Real space

ID	Triangle																				N_W	N_C
t_1	W	W	W	W	C	C	C	C	W	W	W	W	C	W	W	W	C	C	C	W	12	8
t_2	W	C	C	W	C	W	C	C	W	C	C	W	C	C	W	C	C	W	W	C	8	12
t_3	C	C	C	C	C	C	C	W	C	C	C	C	C	C	C	C	C	C	C	C	1	19
t_4	W	C	C	C	C	C	C	C	W	C	C	W	C	C	W	C	C	C	C	W	5	15
t_5	C	C	W	W	C	W	W	C	C	W	C	C	C	W	W	C	W	C	C	W	9	11
t_6	C	C	C	W	C	C	C	W	C	C	W	C	C	C	C	C	C	W	C	C	4	16
t_7	W	W	C	C	W	C	C	W	C	C	C	W	C	W	C	C	W	C	W	W	9	11
ID	Square																				N_W	N_C
t_1	W	W	C	C	W	W	W	W	W	W	C	C	W	W	C	W	W	W	C	C	13	7
t_2	C	C	C	W	W	C	C	C	W	C	W	C	C	W	C	W	C	C	C	W	7	13
t_3	C	C	W	W	C	C	C	W	C	C	W	C	C	C	W	C	C	C	W	C	6	14
t_4	C	C	W	C	C	W	W	W	C	C	C	W	C	C	C	C	W	C	C	C	6	14
t_5	C	W	W	C	C	C	W	C	W	C	C	C	W	C	C	W	C	C	W	C	7	13
t_6	C	C	C	C	C	W	C	C	C	C	W	C	C	C	C	C	C	W	C	C	3	17
t_7	W	W	W	W	W	C	C	W	W	C	C	W	C	C	W	W	C	C	C	W	11	9

Virtual space

ID	Triangle																				N_W	N_C
t_1	W	W	C	C	W	W	W	C	C	W	W	C	C	W	W	C	C	C	W	C	10	10
t_2	W	W	C	W	C	C	W	W	C	C	W	C	C	W	C	C	C	W	C	C	8	12
t_3	C	C	C	C	C	C	C	W	C	C	W	C	C	C	W	W	W	C	C	C	5	15
t_4	W	C	W	W	C	W	C	C	C	W	C	C	W	C	W	W	C	C	C	W	9	11
t_5	C	C	C	W	C	C	W	W	C	C	W	C	C	W	W	C	C	W	C	W	8	12
t_6	C	C	W	C	C	W	W	C	C	C	W	C	C	C	C	C	C	W	C	C	5	15
t_7	C	W	C	C	W	W	C	W	W	C	C	W	W	C	W	W	C	C	W	W	11	9
ID	Square																				N_W	N_C
t_1	C	C	W	C	C	W	W	W	C	C	W	C	C	W	W	W	C	W	W	C	10	10
t_2	C	W	C	W	C	W	C	C	C	C	C	C	C	W	W	C	C	W	C	W	7	13
t_3	C	C	C	C	C	C	C	W	C	C	W	C	C	C	C	C	C	C	C	W	3	17
t_4	C	C	W	W	C	C	W	W	W	C	C	C	W	C	W	W	C	C	W	C	9	11
t_5	C	W	W	C	C	C	C	W	W	C	C	C	W	W	C	C	C	C	W	C	7	13
t_6	W	C	C	C	C	C	C	C	C	C	W	C	C	C	C	W	C	C	C	C	3	17
t_7	W	C	C	W	C	C	C	C	W	W	C	W	W	C	C	W	W	C	W	W	10	10
